# Systematic analysis of the *in situ* crosstalk of tyrosine modifications reveals no additional natural selection on multiply modified residues

**DOI:** 10.1038/srep07331

**Published:** 2014-12-05

**Authors:** Zhicheng Pan, Zexian Liu, Han Cheng, Yongbo Wang, Tianshun Gao, Shahid Ullah, Jian Ren, Yu Xue

**Affiliations:** 1 Department of Biomedical Engineering, College of Life Science and Technology, Huazhong University of Science and Technology, Wuhan, Hubei 430074, China; 2 State Key Laboratory of Biocontrol, School of Life Sciences, Sun Yat-sen University, Guangzhou, Guangdong 510275, China

## Abstract

Recent studies have indicated that different post-translational modifications (PTMs) synergistically orchestrate specific biological processes by crosstalks. However, the preference of the crosstalk among different PTMs and the evolutionary constraint on the PTM crosstalk need further dissections. In this study, the *in situ* crosstalk at the same positions among three tyrosine PTMs including sulfation, nitration and phosphorylation were systematically analyzed. The experimentally identified sulfation, nitration and phosphorylation sites were collected and integrated with reliable predictions to perform large-scale analyses of *in situ* crosstalks. From the results, we observed that the *in situ* crosstalk between sulfation and nitration is significantly under-represented, whereas both sulfation and nitration prefer to co-occupy with phosphorylation at same tyrosines. Further analyses suggested that sulfation and nitration preferentially co-occur with phosphorylation at specific positions in proteins, and participate in distinct biological processes and functions. More interestingly, the long-term evolutionary analysis indicated that multi-PTM targeting tyrosines didn't show any higher conservation than singly modified ones. Also, the analysis of human genetic variations demonstrated that there is no additional functional constraint on inherited disease, cancer or rare mutations of multiply modified tyrosines. Taken together, our systematic analyses provided a better understanding of the *in situ* crosstalk among PTMs.

Through modification of proteins by covalent attachment of other functional groups or by proteolytic cleavage, post-translational modifications (PTMs) temporally and spatially affect protein activity, stability and trafficking, regulate most of biological and physiological functions, and determine the cellular dynamics and plasticity^1^,^2^,^3^. In particular, one PTM can crosstalk with other PTMs to synergistically orchestrate specific processes through three distinct mechanisms^4^,^5^,^6^,^7^,^8^,^9^,^10^,^11^,^12^,^13^. First, different PTMs can co-occur in the same proteins and crosstalk in a *cis*-regulatory manner^4^,^5^. For example, the phosphorylation of the motif ΨKXEXXpSP (Ψ is a hydrophobic residue, X is any amino acid, pS is a phosphorylatable serine) at S303 of HSF1, a heat shock transcription factor, enhances the adjacent lysine sumoylation at K298^4^ (Fig. 1a). Also, a recently computational analysis suggested that a considerable proportion of acetylated lysines might influence the PTMs such as phosphorylation, methylation and ubiquitination of adjacent sites^6^. Second, one PTM can regulate another PTM by modifying its cognate enzymes and *vice versa*, in a *trans*-regulatory mode^7^,^8^,^9^. For example, the E3 ubiquitin ligase complex of Rictor/Cullin-1/Rbx1 ubiquitinates an AGC kinase of SGK1 and promote its degradation, whereas the T1135 of Rictor can be phosphorylated by multiple AGC kinases including SGK1, and such a phosphorylation disrupts the interaction of Rictor and Cullin-1 to inhibit the ubiquitination of SGK1^8^ (Fig. 1b). Third, multiple PTMs can “*in situ*” interplay with each other by competitively modifying same residues^10^,^11^,^12^,^13^. For example, a circadian clock protein PER2 is competitively *O*-GlcNAcylated and phosphorylated at S662, while the *in situ* crosstalk precisely regulates the PER2 repressor activity^13^ (Fig. 1c). In addition, different types of PTM crosstalks can simultaneously occur and regulate biological functions in a complicated manner. For example, PKCδ phosphorylates Caspase-3^14^, which reciprocally cleaves PKC-δ as a *trans*-crosstalk^15^ (Fig. 1d). Also, p53 can be activated through the PKCδ-mediated phosphorylation of S46^16^, and such a phosphorylation promotes its acetylation at K382 through a *cis*-crosstalk^17^ (Fig. 1d). Again, lysine acetylation and methylation compete at K382 for modulating the p53 transcription activity^18^ (Fig. 1d).

Recently, the *in situ* crosstalk of PTMs has emerged to be an intriguing topic and attracted much attention. The first question is whether PTMs recognizing the same type of amino acid residues are significantly co-occurred. For this issue, Wang *et al*. totally characterized 141 O-GlcNAcylation and >350 phosphorylation sites from the mitotic spindle and midbody samples of human HeLa cells, and observed that both PTMs extensively co-occupy at same serine/threonine (S/T) residues or adjacent regions^19^. However, in a following study, Trinidad *et al*. systematically identified ~1750 O-GlcNAcylation and ~16,500 phosphorylation sites from mouse synaptosomes, and revealed that only 8% (135) of O-GlcNAcylation sites are also phosphorylated^20^. With a computational simulation, they observed that the overlap of two PTMs is not significant and nearly equal to be random^20^. The result was supported by an analysis of lysine succinylation and acetylation, in which Park *et al*. detected that only ~22% of succinylation sites were also acetylated at the same lysines from mouse embryonic fibroblasts (MEFs)^21^. However, in a different analysis, Weinert *et al*. demonstrated that succinylation and acetylation extensively co-occupy at same residues from prokaryotes and eukaryotes, whereas 66% of *E. coli*, 56% of *S. cerevisiae*, and 57% of mouse liver succinylation sites are overlapped with acetylation^22^. Interestingly, they also detected that only 27% of succinylation sites are acetylated in human HeLa cells^22^. In this regard, the preference of the *in situ* crosstalk may not only be dependent on the PTM types, but also exhibit a tissue-specific manner. The second and more important question is whether amino acids targeted by multiple PTMs undergo additional evolutionary pressure against singly modified sites. Because the co-occurrence of O-GlcNAcylation and phosphorylation are similar to be random, Trinidad *et al*. suggested that there is little or no evolutionary pressure for the *in situ* crosstalk between two PTMs^20^. In contrast, by analyzing the potentially *in situ* crosstalk of protein lysine modifications (PLMs), although the long-term phylogenetic analysis among multi-species revealed multi-PTM targeting sites are only slightly more conserved than singly modified lysines, the short-term evolutionary results demonstrated that multiply modified lysines dramatically enriched more human disease-associated and rare variations^23^. Thus, Gray *et al*. concluded that mutations of lysines with multi-PTMs undergo much greater purifying selection against singly modified lysine residues^23^.

None of two questions mentioned above have been fully addressed, while different viewpoints were raised for both problems. To further clarify the controversial issues, more efforts should be taken. Recent progresses in the identification of substrates and sites for tyrosine modifications, such as sulfation^24^,^25^,^26^, nitration^27^,^28^ and tyrosine phosphorylation^29^ provided a great opportunity to address the above two problems, as an independent, justified and unbiased framework. Also, previous studies observed that the *in situ* crosstalk truly occurs among the three PTMs. For example, both phosphorylation and nitration can co-occur at Y125, Y133 and Y136 of α-synuclein (UniProt ID: P37840)^30^,^31^. Moreover, human Gastrin (P01350) was identified to be phosphorylated by *v*-Src at Y87^32^, the site which is also modulated by sulfation^33^.

From the scientific literature, public databases and our previous studies^27^, we totally collected 273 sulfation sites, 1,050 nitration sites and 24,242 phosphorylation sites in 171, 539 and 11,034 proteins, respectively. By integrating the datasets together, we observed 2 sulfation-nitration, 3 sulfation-phosphorylation and 183 nitration-phosphorylation site-specific crosstalks (Table S1). Using known sulfation sites as the training data, we developed a novel predictor of GPS-TSP (Tyrosine sulfation predictor, available at http://tsp.biocuckoo.org), which exhibited superior performance than other existed tools by comparison. Together with a nitration sites predictor of GPS-YNO2^27^, we predicted potential nitration sites in sulfated substrates, and *vice versa*. Our results demonstrated the *in situ* crosstalk between sulfation and nitration was significantly under-represented, and two PTMs prefer to regulate different functions. Moreover, by predicting potential sulfation and nitration sites in known phosphorylated substrates, we detected that a considerable proportion of tyrosine phosphorylation sites (24.5%) might be modified by either sulfation or nitration, and both sulfation and nitration preferentially target phosphorylated tyrosines rather than non-phosphorylated sites by 1.71- and 1.45-fold, respectively. Again, statistical results suggested sulfation and nitration prefer to crosstalk with phosphorylation in regulating potentially distinct biological processes and functions. Further analyses of the sequence and structure preferences revealed that different types of tyrosine modifications prefer to co-occur at distinct structural positions in proteins. Interestingly, whether PTMs prefer to *in situ* crosstalk or not has no correlation with the evolutionary constraint on multiply modified tyrosines. In our results, although three types of tyrosine modifications have distinct preferences for the *in situ* crosstalk, the long-term evolutionary analysis across eight vertebrate species revealed that multiply modified tyrosines are not more conserved than unmodified ones. In addition, by mapping all known or predicted tyrosine modification sites to human genetic variations, we observed that multiply modified tyrosines didn't significantly enrich inherited disease, cancer or rare mutations. Taken together, our results suggested that there is no functional constraint on multiply modified tyrosines, and the *in situ* crosstalk of tyrosine modification does not need additional natural selection.

## Results

### Development of GPS-TSP for the prediction of sulfation sites

**Figure 2:**
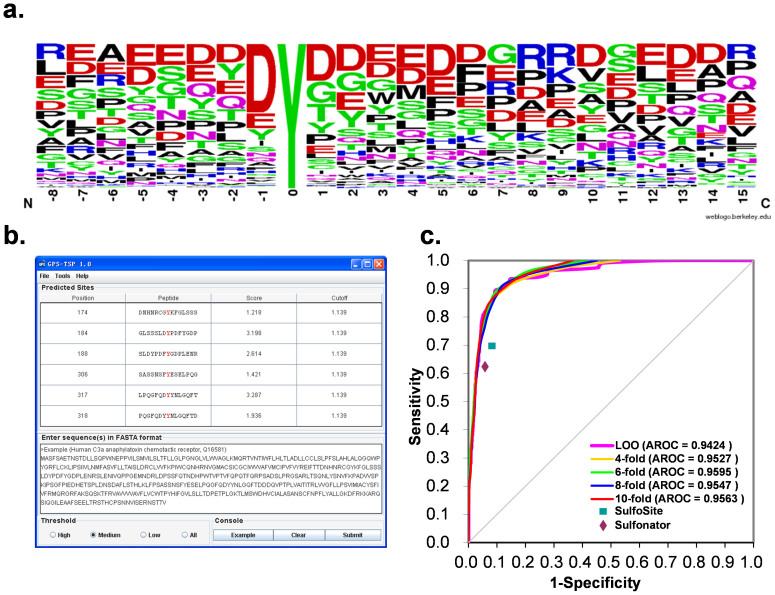
The development of GPS-TSP 1.0. (a) The sequence logo of sulfation sites. (b) The snapshot of GPS-TSP with the example of human C3a complement anaphylatoxin chemotactic receptor (C3aR, Q16581). (c) The ROC curves and AROC values for the LOO validation and 4-, 6-, 8-, 10-fold cross-validations.

For systematically analyzing the tyrosine sulfation and its crosstalk with other tyrosine modifications, a prerequisite is to establish a comprehensive and reliable dataset. However, the experimental identification of sulfation substrates is still labor-intensive and time-consuming, while only 273 known sulfation sites were collected. Thus, computational prediction of sulfation sites from protein primary sequences can serve as an alternative solution. Before developing the tool, we first analyzed the sequence profile of 202 non-redundant sulfation sites by WebLogo^34^ (Fig. 2a). Although a previous analysis proposed that the +2 position didn't contain any information with a limited data set^26^, our analysis clearly exhibited that negatively charged residues including aspartic acids and glutamic acids were enriched around the sulfated tyrosine especially the position -1, while tyrosine was founded to be over-presented in position +1, +2, and upstream positions. In addition, glycine and glutamine were observed to frequently occur near the sulfated tyrosine. These observations suggested that there were considerable sequence preferences around sulfation sites (Fig. 2a). Then we designed a software package of GPS-TSP for predicting tyrosine sulfation sites, with a previously developed algorithm of Group-based Prediction System (GPS)^27^ (Fig. 2b). More details on the algorithm were shown in Supplemental experimental procedures.

To evaluate the prediction performance and robustness, the LOO validation and 4-, 6-, 8-, 10-fold cross-validations were performed. The corresponding ROC curves were presented, while the AROC values were calculated as 0.9424 (LOO), 0.9527 (4-fold), 0.9595 (6-fold), 0.9547 (8-fold) and 0.9563 (10-fold), respectively (Fig. 2c). Since the results of the 4-, 6-, 8- and 10-fold cross-validations were closely similar to the LOO validation, the prediction is evidently stable and robust. The performance of the LOO validation was used for the cut-off setting and further comparison, and the three thresholds of high, medium and low were selected with the *Sp* values of 85%, 90% and 95%, respectively (Table 1).

Previously, a number of computational studies were performed for predicting protein sulfation sites, however, only Sulfinator^35^ and SulfoSite^36^ were implemented into online services. To demonstrate the superiority of GPS-TSP, here we used its training dataset of 202 sulfation sites and 1027 negative sites to evaluate the performances of two predictors. To avoid any bias, we compared the *Sn* values for GPS-TSP, Sulfinator and SulfoSite at the equal level of *Sp* values (Table 1, Fig. 2c). When the *Sp* value was ~93%, the *Sn* value of GPS-TSP and Sulfinator were 83.17% and 61.79%, respectively (Table 1). Also, when the *Sp* value was ~91%, the *Sn* of GPS-TSP (87.12%) was much greater than SulfoSite (69.73%) (Table 1). In this regard, the prediction performance of GPS-TSP 1.0 is much better than other existed tools.

### Sulfation and nitration prefer not to co-occur at same tyrosines

Besides sulfation, protein tyrosine nitration (PTN) is also an important PTM, and predominantly implicated in a variety of fundamental processes such as RNA splicing, mRNA processing and translation^27^,^28^, whereas sulfation was proposed to mostly occur in the secretory pathway^24^,^25^,^26^. To further clarify whether two PTMs prefer to target and regulate distinct processes and functions, the experimentally identified nitrated substrates were taken from our previous study^27^. In order to analyze and compare the functional abundance and diversity of sulfation and PTN, we downloaded the gene ontology (GO) (March 31^th^, 2012) association files from the GOA database at the EBI (http://www.ebi.ac.uk/goa)^37^. There were 44,741 human proteins, 65 sulfated proteins and 326 nitrated proteins annotated with at least one GO term.

**Figure 3:**
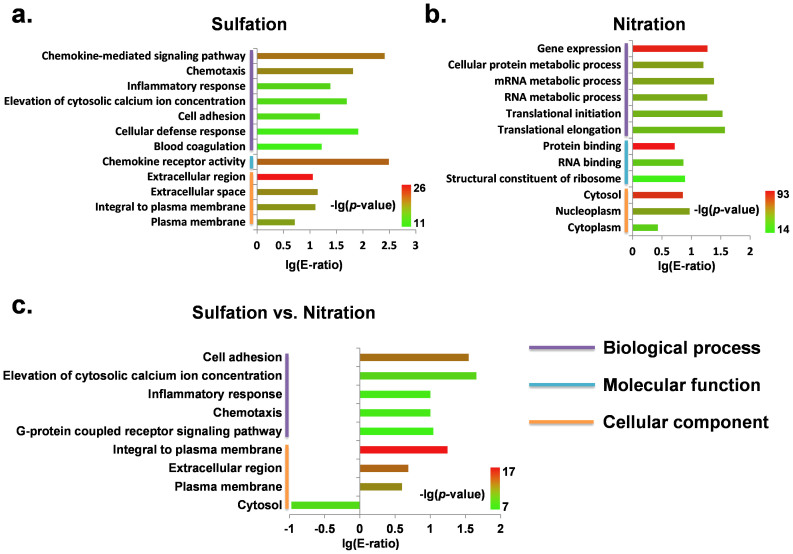
Statistical analyses of GO annotations for sulfated and nitrated proteins. The enriched GO terms for sulfated proteins (a) or nitrated proteins (b) in comparison with proteome. (c) Comparison of GO terms between sulfated and nitrated proteins. E-ratio, enrichment ratio.

With the hypergeometric distribution^27^, we statistically analyzed the enriched biological processes, molecular functions and cellular components with GO annotations for sulfated (Fig. 3a, *p*-value < 10^−11^) and nitrated (Fig. 3b, *p*-value < 10^−14^) proteins. For the sulfated substrates, the top five most enriched biological processes are chemokine-mediated signaling pathway (GO:0070098), chemotaxis (GO:0006935), inflammatory response (GO:0006954), elevation of cytosolic calcium ion concentration (GO:0007204), and cell adhesion (GO:0007155), which are consistent with previously experimental observations^24^,^25^,^26^ (Fig. 3a). However, the top five most significant processes of nitration are gene expression (GO:0010467), cellular protein metabolic process (GO:0044267), mRNA metabolic process (GO:0016071), RNA metabolic process (GO:0016070), and translational initiation (GO:0006413)(Fig. 3b). In this regard, we proposed that sulfation and nitration are preferentially involved in distinct processes.

To confirm this analysis, we compared the functional diversity of sulfated and nitrated proteins using the Yates' Chi-square (χ^2^) test^27^ (Fig. 3c, *p*-value < 10^−7^). Indeed, sulfation was found to be preferentially involved in modifying membrane and extracellular proteins, while nitration prefers to attack substrates in cytosol (Fig. 3c). In addition, we used the high thresholds of GPS-TSP and GPS-YNO2^27^, and directly predicted potential nitration sites from sulfated substrates and *vice versa* (Table 2). With the hypergeometric distribution, the results clearly demonstrated that sulfation and nitration prefer not to *in situ* interplay at the same positions (Table 3).

### Sulfation and nitration prefer to *in situ* crosstalk with tyrosine phosphorylation

To further dissect the relations among tyrosine modifications, the *in situ* crosstalk between sulfation or nitration and phosphorylation were surveyed. We used GPS-TSP and GPS-YNO2 with the high thresholds to predict potential sulfation and nitration sites in tyrosine phosphorylated substrates (Table 3 & Table S4). Totally, it was observed that 5,939 (24.5%) known phosphorylation sites might be modified by either sulfation (2,913, 12.0%) or nitration (3,689, 15.2%) (Table S2). Although the *p*-values in *D. melanogaster* and *C. elegans* were not much significant due to the data limitation, the statistical results suggested that sulfation and nitration prefer to occur at phosphorylated tyrosines rather than non-phosphorylated tyrosines with the enrichment ratios (E-ratios) of 1.71 and 1.45, respectively (Table 3). Because our dataset of tyrosine phosphorylation contained sites identified from large-scale studies which couldn't guarantee all data to be real phosphorylation sites, we further obtained 3,254 well curated tyrosine phosphorylation sites from Phospho.ELM (version 9.0, released in April 2010)^38^. Again, the results still suggested that sulation and nitration prefer to *in situ* crosstalk with phosphorylation (Table S3).

**Figure 4:**
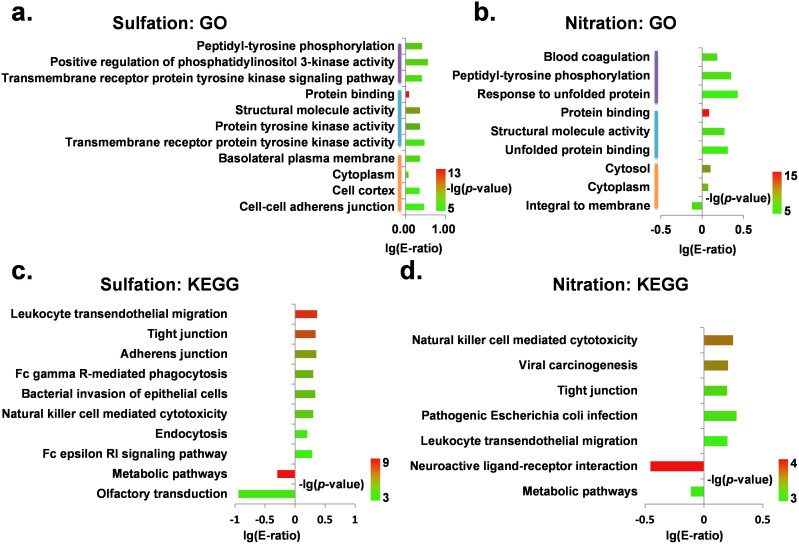
Statistical analyses of GO and KEGG annotations for proteins with *in situ* crosstalk between sulfation or nitration and phosphorylation. The enriched GO terms for proteins with *in situ* crosstalk between sulfation (a) or nitration (b) and phosphorylation in comparison with phosphorylated proteins. The enriched KEGG annotations for proteins with *in situ* crosstalk between sulfation (c) or nitration (d) and phosphorylation in comparison with phosphorylated proteins.

With the hypergeometric distribution, we statistically analyzed the over- or under-represented GO terms in phosphorylated substrates which might also be competitively regulated by sulfation (Fig. 4a, *p*-value < 10^−5^) or nitration (Fig. 4b, *p*-value < 10^−5^) at the same residues, separately. We only considered the predicted sulfated and nitrated proteins, whose sites were predicted from known phosphorylation sites. Clearly, sulfation prefers to *in situ* crosstalk with phosphorylation in a variety of biological processes, such as peptidyl-tyrosine phosphorylation (GO:0018108), positive regulation of phosphatidylinositol 3-kinase activity (GO:0043552), and transmembrane receptor protein tyrosine kinase signaling pathway (GO:0007169) (Fig. 4a). In contrast, nitration prefers to *in situ* crosstalk with phosphorylation in blood coagulation (GO:0007596), peptidyl-tyrosine phosphorylation (GO:0018108), and response to unfolded protein (GO:0006986) (Fig. 4b). By comparison, it was observed that sulfation and nitration prefer to *in situ* crosstalk with phosphorylation in distinct biological processes and functions. Moreover, we mapped all phosphorylated proteins to the Kyoto Encyclopedia of Genes and Genomes (KEGG) pathways^39^, and performed the statistical analyses for the *in situ* crosstalks. The results suggested that the sulfation-phosphorylation crosstalk is significantly enriched in pathways of leukocyte transendothelial migration (hsa04670), tight junction (hsa04530), and adherens junction (hsa04520) (Fig. 4c), whereas the nitration-phosphorylation crosstalk prefers to target natural killer cell mediated cytotoxicity (hsa04650), viral carcinogenesis (hsa05203), and tight junction (hsa04530) (Fig. 4d). Taken together, although both sulfation and nitration prefer to co-occupy with phosphorylation, the sulfation-phosphorylation and nitration-phosphorylation crosstalks may preferentially occur in different pathways.

### The sequence and structure preferences of the tyrosine modifications

**Figure 5:**
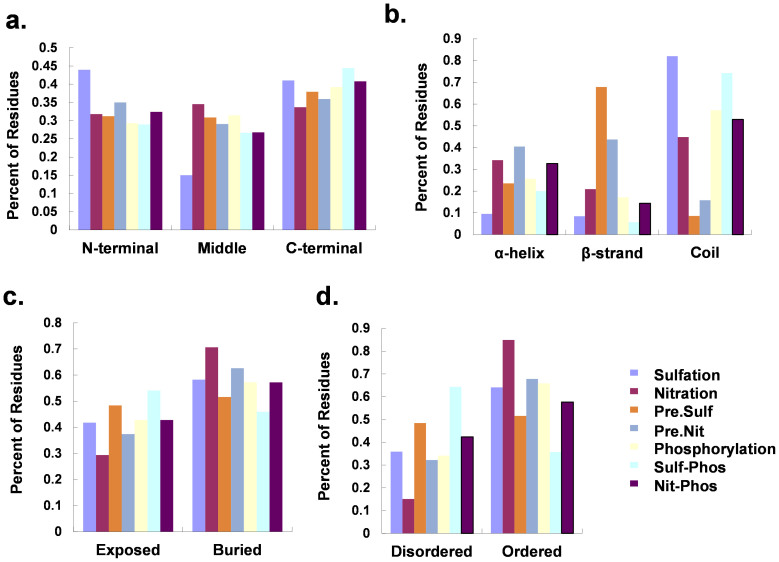
The sequence and structure preferences of known or predicted tyrosine modification sites. Sulfation, Nitration, and Phosphorylation denote known modified tyrosines. Pre. Sulf., predicted sulfation sites in phosphorylated proteins; Pre. Nit., predicted nitration sites in phosphorylated proteins; Sulf.-Phos., *in situ* crosstalk of sulfation and phosphorylation in phosphorylated proteins; Nit.-Phos., *in situ* crosstalk of nitration and phosphorylation in phosphorylated proteins. (a) Position distribution of modified tyrosines in N-terminal, Middle, or C-terminal regions in protein sequences. (b) Distribution of modified tyrosines in a-helix, β-Strand, Coil of the secondary structure. (c) Distribution of tyrosine modification residues in exposed and buried regions. (d) Distribution of modified tyrosines in disordered and ordered regions.

Since the relations among sulfation, nitration and phosphorylation are complicated, we further analyzed the sequence and structure preferences of the tyrosine modifications for dissecting the basic features of the *in situ* crosstalks. The experimentally identified PTM tyrosines including 273 sulfation, 1050 nitration and 24,242 phosphorylation sites were employed for the analysis. Furthermore, 17,015 and 25,306 predicted sulfation and nitration sites in phosphorylated substrates were also analyzed, while 2913 and 3689 phosphorylation sites were predicted to be sulfation- and nitration-phosphorylation crosstalk sites. With these datasets, we first analyzed the position distributions of modified tyrosines. In the result, known sulfation sites preferentially occur at N-terminal or C-terminal but not middle of proteins, whereas predicted sulfation sites slightly prefer to locate at C-terminal, as well as other types of tyrosine modifications and their crosstalks (Fig. 5a).

Also, various structural features were analyzed. From the results of secondary structures, it was observed that both known sulfation and nitration sites are enriched in Coil, whereas predicted nitration sites are deprived in Coil and sulfation sites are enriched in β-Strand (Fig. 5b). This might be due to the number of known sulfation and nitration sites is still quite limited, and the secondary structural preferences of two PTMs remain to be further characterized when more sites are identified. Interestingly, although both PTMs prefer not to occur at Coil, they significantly co-occupy with phosphorylation sites at Coil (Fig. 5b). Moreover, from the analysis of the protein surface accessibility, although three types of tyrosine modifications prefer to target buried tyrosines, the result suggested that sulfation-phosphorylation crosstalk preferentially co-occur at exposed tyrosines (Fig. 5c). This result is quite similar with a following analysis of protein disordered regions, in which we revealed that all three PTMs prefer to locate at protein ordered regions. Although the *in situ* crosstalk of nitration and phosphorylation also prefers to localize at ordered tyrosines, the sulfation-phosphorylation crosstalk preferentially target disordered tyrosine residues (Fig. 5d). Taken together, our analyses suggested that sulfation and nitration *in situ* crosstalk with phosphorylation prefer to occur at distinct structural positions in proteins.

### The long-term evolutionary analysis revealed almost no additional natural selection of multiply modified tyrosines

**Figure 6:**
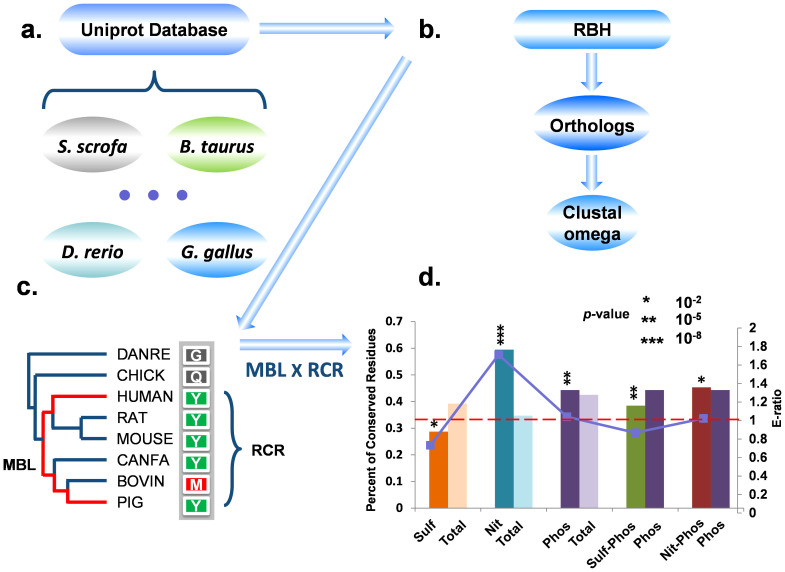
The computational procedure for analyzing the long-term evolution of tyrosines. (a) Proteome sets of eight vertebrates were obtained from the UniProt database^55^. (b) The orthologs were pairwisely detected^42^, and the multiple sequence alignment was performed by Clustal Omega^40^. (c) The *RCS_Y_* is calculated by the number of tyrosines appearing in the column of multi-alignment of orthologs where the species with the MBL containing the tyrosine residues^43^. (d) The conservation levels of different types of modified or unmodified tyrosines. The lines with dots represent the enrichment ratio between the two datasets. Total, total tyrosines.

With experimentally identified PTM sites, we directly detected 2 sulfation-nitration, 3 sulfation-phosphorylation and 183 nitration-phosphorylation site-specific crosstalks (Table S1). In particular, there are ~82% (154) of total *in situ* crosstalks in *H. sapiens* (Table S1). Thus, the long-term evolutionary analysis was only performed for human tyrosine modifications, due to the data limitation. Totally, we took 101 sulfotyrosines of 880 total tyrosines in 48 known sulfated proteins, 564 nitrotyrosines of 5233 tyrosines in 328 known nitrated substrates, and 13,730 phosphotyrosines of 126,147 tyrosine residues in 5876 known phosphorylated proteins from *H. sapiens*. Also, 1604 and 2051 predicted sulfation and nitration sites on 13,730 known human phosphorylation sites were considered. Because the phosphoregulation and phosphoproteome rapidly evovle^40^,^41^, here we focused on analyzing the potentially natural selection of tyrosines after the speciation of vertebrates. We obtained the proteome sets of several other vertebrates from the UniProt database (Fig. 6a). Then we computed pairwise orthologs among these species, and further multi-aligned the orthologous proteins together for each cluster of orthologous groups (COGs)^42^ (Fig. 6b). As previously described^43^, we calculated *RCS_Y_* values of modified and unmodified tyrosines, and only residues with *RCS_Y_* = 1 were regarded as conserved tyrosines (Fig. 6c).

In our results, phosphorylation sites are slightly more conserved than unmodified tyrosines (*p*-value < 10^−5^), and the ratio of modified/unmodified site (E-ratio) was only 1.04 (Fig. 6d). The result was consistent with previous studies, which demonstrated that the conservation of phosphorylated and non-phosphorylated tyrosines is quite similar^40^,^44^. However, we further observed that known nitration sites are significantly more conserved than non-nitrated ones (Fig. 6d, *p*-value < 10^−8^, E-ratio = 1.71), and the result was also consistent with a previous report^45^. In contrast, sulfation sites are significantly less conserved than unmodified tyrosines (Fig. 6d, *p*-value < 0.01, E-ratio = 0.73). In this regard, the conservation of distinct tyrosine modifications is quite different. Adding more or less species for the evolutionary analysis didn't influence the results, as well as changing the *RCS_Y_* threshold from 5/8 to 1.

Furthermore, we analyzed the conservation of sulfation-phosphorylation and nitration-phosphorylation crosstalk sites against known phosphorylation sites (Fig. 6d). Unexpectedly, the tyrosines modified by both sulfation and phosphorylation are statistically less conserved than phosphorylation sites (*p*-value < 10^−5^, E-ratio = 0.87). Although nitration-phosphorylation crosstalk sites look like to be significantly more conserved than phosphorylation sites, the E-ratio of crosstalk/phosphorylation sites was only 1.02 (Fig. 6d, *p*-value < 0.01). In this regard, the evolutionary pressure on tyrosines with nitration and phosphorylation is extremely weak against phosphorylation sites, and no signature of additional natural selection was observed for the sulfation-phosphorylation crosstalk. This might be due to tyrosine residues undergo stronger purifying selection than other types of amino acids, and both phosphorylated or non-phosphorylated tyrosines slowly evolve with a similar rate^40^,^44^. Thus, additionally evolutionary pressure is not necessary or difficult to be detected. Again, the change of either the number of species or the *RCS_Y_* threshold didn't influence the results (Data not shown).

### The analysis of human genetic variations revealed no functional constraint on tyrosines with multiple PTMs

As previously described^23^, we mapped all experimentally identified and computationally predicted tyrosine modification sites in this study to the HGMD database^46^, but only detected 2 sulfation-phosphorylation and 6 nitration-phosphorylation crosstalk sites that are associated with human inherited diseases (Table S4). We also mapped these sites to the CanProVar database^47^, but still only obtained 3 sulfation-phosphorylation and 4 nitration-phosphorylation crosstalk sites that are potentially implicated in human cancers (Table S5). Again, we mapped the sites to human rare variations (allele frequency < 1%) (Table 4)^23^. The statistical results suggested that neither sulfation-phosphorylation nor nitration-phosphorylation crosstalk sites could significantly enrich more disease-associated or rare variations (Table 4). Indeed, human genetic variations or mutations will unambiguously disrupt the *in situ* crosstalks of tyrosine modifications for the specific sites, but no additionally functional constraint was observed for the crosstalks.

## Discussion

Accumulative studies have exhibited the ubiquity and importance of PTM crosstalks^5^,^6^,^7^,^9^. By reciprocally modifying upstream enzymes^7^,^9^, by modifying amino acids to change the modification states of adjacent sites^5^,^6^, or by competitively regulating the same residue^10^,^11^,^12^,^19^,^30^,^31^,^32^,^33^, various PTMs can precisely orchestrate specific biological processes by crosstalks. In this regard, experimental identification of PTM crosstalks is fundamental for understanding the complexities and regulatory mechanisms of PTMs.

With GPS-YNO2^27^ and a newly developed tool of GPS-TSP, we directly predicted potential nitration sites in sulfated proteins and *vice versa*. The statistical results demonstrated that *in situ* crosstalk between sulfation and nitration was significantly under-represented (Table 2). This observation might be associated with the different involvements of pathways for sulfation and nitration. However, different sequence preferences might be the basic cause of the low overlap between the two PTMs. By comparing GO terms of sulfated and nitrated proteins, we observed that most of significantly different GO terms are in sulfation (Fig. 3c). Thus, we proposed that the regulation of sulfation is much more specific than nitration. Also, the performance of GPS-YNO2 for the prediction of nitration sites is limited as 87.51% of *Ac*, 71.33% of *Sn*, and 89.84% of *Sp*^27^, while the performance of GPS-TSP with the default threshold is 90.23% of *Ac*, 89.60% of *Sn*, and 90.36% of *Sp* (Table 1). Thus, the sequence profile of sulfation is much more stringent than nitration.

By computationally predicting sulfation and nitration sites in known tyrosine phosphorylated substrates, we observed that up to 24.5% of known phosphorylation sites might also be modified by either sulfation or nitration, while statistical results suggested that both sulfation and nitration prefer to *in situ* crosstalk with phosphorylation at the same phosphorylation sites rather than non-phosphorylatable tyrosines (Table 3, Table S2 & S3). Interestingly, the overlapping rate between sulfation and nitration for the crosstalk with phosphorylation is quite low. Thus, the results suggested that sulfation and nitration preferentially crosstalk with phosphorylation in distinct biological processes and functions (Fig. 4). Notably, our analysis of GO terms demonstrated that sulfation and phosphorylation moderately prefer to co-occur at the same residues of proteins located in cytoplasm (GO:0005737) (Fig. 4a, *p*-value <10^−5^, E-ratio = 1.19). The result seems conflicting with known evidence that sulfation exclusively occurs in secreted pathways because two tyrosylprotein sulfotransferases, TPST-1 and TPST-2, locate in the trans-Golgi network, and almost all of sulfated proteins were identified in extracellular or membrane-related regions^24^,^25^,^26^. Actually, from the 42 human sulfated substrates manually curated in this study, it was observed that only 5 proteins localized exclusively in extracellular positions, while 20 proteins have no extracellular localizations. Our hypothesis was that the proteins with sulfation-phosphorylation crosstalks might also locate at other subcellular localizations beyond cytoplasm. Indeed, after we removed proteins with additional GO terms on golgi, extracellular or membrane in both phosphorylated and crosstalk substrates, the statistical significance didn't exist any longer (*p*-value > 0.05).

Undoubtedly, our results will be helpful for further experimental consideration. For example, the molecular masses of a nitro group (·NO_2_) is ~46 Da, while the phosphate and sulfate groups are 79.9663 and 79.9568 Da, respectively^48^. In this regard, unambiguously distinguishing tyrosine phosphorylation and sulfation is particularly difficult and error-prone by currently experimental approaches. Indeed, the human trypsin 1 (UniProt ID: P07477) was first identified to be phosphorylated at Y151^49^, whereas a later experiment verified that the site is sulfated^50^. Although it's not possible that nearly 2900 phosphorylation sites are actually sulfated (Table 3), great attention should be paid to experimentally identifying either phosphorylation or sulfation sites, given the preference of *in situ* crosstalk between sulfation and phosphorylation. Since currently systematical profiling of sulfation sites is yet to be carried out, the predictions based on the limited small-scale experimental data could serve as a start for the studies of crosstalks between sulfation and phosphorylation. In addition, because the large-scale data set of tissue-specific sulfation or nitrations sites is still not available, whether the preference of in situ crosstalks of tyrosine modifications will be changed in different tissues or organs remains to be dissected. However, our current results still clearly demonstrated that different types of tyrosine modifications can have distinct preferences for the *in situ* crosstalk.

In his classical essay, Theodosius Dobzhansky stated “nothing in biology makes sense, except in the light of evolution”^51^. Thus, the analysis of PTMs should be performed under the framework of evolutionary biology. Indeed, a number of evolutionary studies have characterized additional natural selection on the phosphorylation and phosphoproteome against unmodified residues, although only a weak evolutionary pressure was observed^40^,^41^,^44^. Recently, more controverisal veiwpoints have emerged toward the residues with multiple PTMs, and whether the *in situ crosstalk* of PTMs with an additionally evolutionary pressure still remains to be elusive^19^,^20^,^21^,^22^,^23^. In contrast with previous studies that the evolutionary analyses were performed at the genome- or proteome-wide level^40^,^41^,^44^, Gray *et al*. defined a protein-specific measure, α, to estimate the long-term natural selection on individual proteins^23^,^52^. Although such a new approach was used, the analysis demonstrated that lysines with multiple PTMs are slightly more conserved than singly modified lysines, with only a 6% difference^23^. Also, our long-term evolutionary analysis of the *in situ* crosstalks among tyrosine modifications revealed that only nitration-phosphorylation but not sulfation-phosphorylation crosstalk sites are slightly more conserved than phosphorylation sites, with a very small E-ratio of 1.02 (Fig. 6d). In this regard, although the statistical significance exists in the previous^23^ and our results, only very weak to no evolutionary pressure was detected for multiply modified sites from the long-term analyses.

For the short-term evolutionary analysis, Gray *et al*. mapped 37,720 singly and 3961 multiply modified lysines to disease-associated mutations in the HGMD database^46^, and only 104 (0.28%) and 17 (0.43%) variations were mapped to lysines with single and multiple PLMs, respectively^23^. Also, we found 2 (0.12%) sulfation- and 6 (0.29%) nitration-phosphorylation crosstalk sites occur at HGMD mutations (Table 4). Thus, only an extremely small proportion of singly or multiple PTM sites are associated with human inherited diseases, whatever the statistical value is significant or not. Moreover, Gray *et al*. hypothesized that if additional negative selection exists in multiply modified lysines, they would prefer to occur at variations with lower allele frequencies. Indeed, the fractions of rare variations (allele frequency < 1%) for unmodified, singly, doubly and triply modified lysines are 88.8%, 91.1%, 93.8% and 100%, respectively^23^. For tyrosine modifications, we only detected one common variation (allele frequency > 1%) on phosphorylation sites, and didn't find any other common variations on crosstalk sites. Thus, it seems that residues with multiple PTMs prefer to occur at rare variations and undergo additional evolutionary pressure. To draw this conclusion, a prerequisite is that multiply modified with multiple PTMs can really enrich the human variations or mutations. However, our results on variations in the HGMD^46^, GWASdb^53^ and 1000 Genomes project^54^ demonstrated that human variations were not over-represented in multiply modified tyrosines (Table 4). Taken together, no additional evolutionary pressure was detected on sites with multiple PTMs from the short-term analysis.

More interestingly, although no evolutionary pressure was detected for the *in situ* crosstalk of O-GlcNAcylation and phosphorylation because of nearly random co-occurrence^20^, we didn't detect any additional natural selection on significantly co-occupied sulfation- and nitration-phosphorylation crosstalk sites. Thus, whether different PTMs prefer to *in situ* crosstalk or not, little or no additional evolutionary pressure exists on residues with multiple PTMs against singly modified sites.

## Methods

### The data sets of tyrosine modifications

By searching the PubMed with multiple keywords such as “tyrosine sulfation”, “sulfation” and “sulfated”, we collected 273 experimentally identified tyrosine sulfation sites in 171 proteins, and integrated the dataset into the DOSS database (http://tsp.biocuckoo.org/database.php). The protein sequences were retrieved from the UniProt database^55^. As previously described^27^, we regarded the known sulfation sites as positive data (+), while other non-sulfated tyrosines were taken as negative data (-). Because the redundancy of homologous sites in the positive data (+) leads to overestimated prediction, we used CD-HIT (http://weizhong-lab.ucsd.edu/cd-hit/) to cluster the protein sequences^56^, followed by re-alignment with BLAST packages and manual check of proteins with ≥40% identify^57^. The redundant sulfation sites at the same position in the homologous proteins according to the alignment results were cleared. Finally, a non-redundant dataset for training was constructed with 202 positive sites and 1,027 negative sites from 116 substrates. The manually collected dataset and the non-redundant training dataset were provided for download at http://tsp.biocuckoo.org/down.php.

For the analysis of the *in situ* crosstalk among sulfation, nitration and phosphorylation, we took 1050 known nitration sites of 539 proteins from a previously published study^27^. The experimentally identified phosphorylation sites were taken from several major databases, including PhosphoPep v2.0^58^, Phospho.ELM 8.3 (released in April 2010)^38^, SysPTM 1.1^59^, PhosphoSitePlus^60^, and HPRD 9.0^61^. We obtained 5876, 4302, 657 and 199 tyrosine phosphoproteins from *H. sapiens*, *M. musculus*, *D. melanogaster* and *C. elegans*. The phosphorylation information in *S. cerevisiae* was not used, because experimental efforts suggested there is not sulfation in yeast^3^,^62^. Totally, 24,242 tyrosine phosphorylation sites were collected from 11,034 proteins, which were also available for download at http://tsp.biocuckoo.org/down.php.

### Performance evaluation

To evaluate the performance of GPS-TSP for the prediction of tyrosine sulfation sites, four measurements of accuracy (*Ac*), sensitivity (*Sn*), specificity (*Sp*), and Mathew Correlation Coefficient (*MCC*) were adopted and defined as below^27^,^63^: 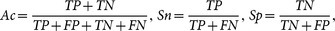
and 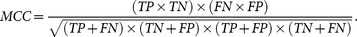


The leave-one-out (LOO) validation and 4-, 6-, 8- and 10-fold cross-validations were carried out. The Receiver Operating Characteristic (ROC) curves and AROC (area under ROC) values were also calculated.

### The sequence and structure analysis

To assess the position distribution of modified tyrosine residues in protein sequences, we equally separated a protein into three fragments, namely, N-terminal, Middle and C-terminal, and directly counted the number of residues in each part. The secondary structures were predicted NetSurfP server ver. 1.1 (http://www.cbs.dtu.dk/services/NetSurfP/), which calculates a probability score for each of three major types of secondary structures including α-Helix, β-Strand and Coil, respectively^64^. The secondary structure of a modified tyrosine was assigned into the type with the highest probability score. Also, NetSurfP predicts both Relative Surface Accessibility (RSA) and Absolute Surface Accessibility (ASA), and tyrosines were classified as buried or exposed based on a threshold of 25% exposure^64^. Moreover, the disordered regions in proteins were directly predicted by RONN (https://www.strubi.ox.ac.uk/RONN)^65^.

### The long-term evolutionary analysis

To analyzed the conservation of modified and unmodified tyrosines in *H. sapiens*, we further downloaded 41,118, 17,864, 28,853, 51,192, 26,457, 24,214, and 26,138 proteins for *D. rerio* (DANRE), *G. gallus* (CHICK), *R. norvegicus* (RAT), *M. Musculus* (MOUSE), *C. familiaris* (CANFA), *B. Taurus* (BOVIN), and *S. scrofa* (PIG) from the UniProt database (November, 2013)^55^, respectively. As previously described^42^, the approach of reciprocal best hits (RBHs) was used by pairwisely detecting orthologs among the eight species, and the blastall program in the BLAST package was chosen. All orthologous proteins among different species were multi-aligned by Clustal Omega (http://www.clustal.org/omega/)^40^. In a multiple sequence alignment (MSA), if a human tyrosine is sulfated, nitrated and/or phosphorylated, the corresponding column was regarded as the modified position. Other columns containing human tyrosines were taken as the unmodified positions. To calculate the conservation of a human tyrosine residue from a MSA, we adopted a previously reported measurement of Residue Conservation Score (RCS), which is expressed as Residue Conservation Ratio (RCR)^43^. Given a tyrosine column, the *RCS_Y_* of can be calculated as below: 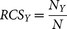


The *N* is the number of sequences with the maximum branch length (MBL), which is the maximum branch distance between any two species that contain a conserved tyrosine. The *N_Y_* is the number of tyrosines appears in the column. The tyrosine residues with RCS_Y_ = 1 were regarded as conserved tyrosines. The change of the RCS_Y_ threshold (0.625 ~ 1) didn't influence the final results. The species tree of the eight organisms were taken from the Interactive Tree Of Life (iTOL, http://itol.embl.de/)^40^.

### The data sets of human genetic mutations and variations

We downloaded 153,839 human disease-associated single nucleotide polymorphisms (SNPs) from the GWASdb^53^. Also, the human inherited disease-associated mutations with annotated information of the Human Gene Mutation Database (HGMD)^46^ were obtained from the ClinVar dataset (March 20^th^, 2012) in the NCBI ftp server, including 78,827 records with 15,641 non-redundant missense mutations. Moreover, we downloaded 73,270 cancer-associated variants from the CanProVar database (September 20^th^, 2012)^47^. In addition, the SNPs of the 1000 Genomes project were downloaded (June 1^st^ 2013, phase I release v3)^54^. Totally, we obtained 325,159 coding SNPs (cSNPs) with reference allele frequencies from four ancestry-based super population groups (AMR, Ad Mixed American; ASN, East Asian; AFR, African; EUR, European).

## Author Contributions

Y.X. designed and supervised experiments. Z.P. and Z.L. performed experiments and data analysis. H.C., Y.W., T.G., S.U. and J.R. contributed to data analysis. Y.X., Z.P. and Z.L. wrote the manuscript with contributions of all authors.

## Supplementary Material

Supplementary Information Supplemental experimental procedures and supplemental tables

## Figures and Tables

**Figure 1:**
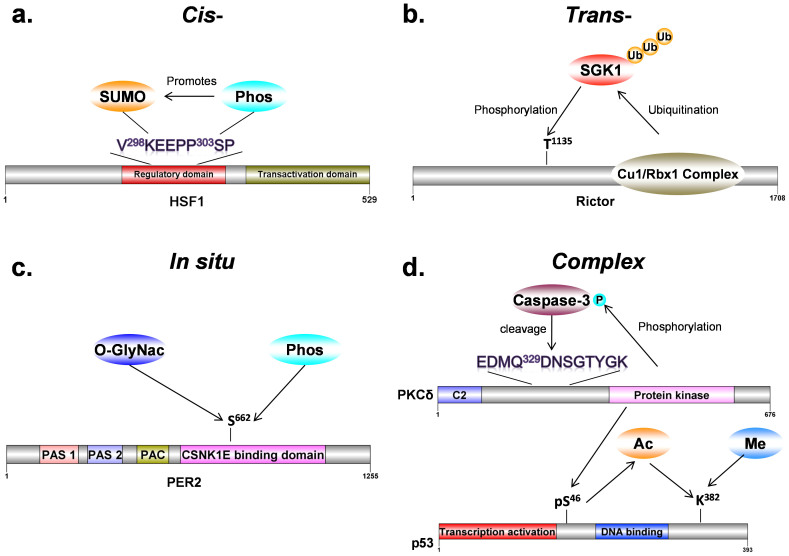
The examples for different types of PTM crosstalks. (a) *Cis-*crosstalk, phosphorylation at S303 of HSF1 promotes its K298 sumoylation^4^. (b) *Trans-*crosstalk between ubiquitination of SGK1 by Rictor/Cullin-1/Rbx1 and phosphorylation of Rictor by SGK1^8^. (c) *In situ* crosstalk, protein PER2 is competitively *O*-GlcNAcylated and phosphorylated at S662^13^. (d) A complex crosstalk among PKCδ, Caspase-3, and p53 that different types of PTM crosstalks can simultaneously occur and regulate biological functions.

**Table 1:** Comparison of the GPS-TSP with other tools. For construction of the software package, the three thresholds of high, medium and low were chosen, while the medium threshold was selected as the default cut-off value. For comparison, we fixed the *Sp* values of GPS-TSP so as to be similar or identical to the other methods and compared the *Sn* values

Tool	Threshold	*Ac*	*Sn*	*Sp*	*MCC*
GPS-TSP	High	92.60%	79.70%	95.13%	0.7354
	Medium	90.23%	89.60%	90.36%	0.7066
	Low	86.57%	93.56%	85.20%	0.6519
		91.54%	83.17%	93.18%	0.7161
		90.48%	87.12%	91.13%	0.7034
Sulfonator		87.63%	61.79%	93.02%	0.5588
SulfoSite		88.45%	69.73%	91.09%	0.5403

**Table 2:** Predicted nitration sites in sulfated substrates and *vice versa*. The hypergeometric distribution was adopted. *a*. The number of known sulfated or nitrated substrates; *b*. The number of all tyrosine residues; *c*. The number of known sulfation or nitration sites; *d*. The number of totally predicted nitration or sulfation sites; *e*. The number of predicted nitration sites on known sulfation residues, and *vice versa*

	Known			Predicted			
PTM	Sub.*^a^*	Total*^b^*	Site*^c^*	Total*^d^*	Site*^e^*	E-ratio	*p*-value
**Sulfation**	171	1,518	273	525	68	0.83	1.02E-03
**Nitration**	539	8,356	1,050	549	46	0.67	8.67E-04

**Table 3:** Predicted sulfation and nitration sites on known phosphorylated substrates, which were collected from *H. sapiens* (HS), *M. musculus* (MM), *D. melanogaster* (DM) and *C. elegans* (CE). The hypergeometric distribution was adopted. *a*. The number of all tyrosine residues; *b*. The number of known phosphorylation sites; *c*. The number of totally predicted sulfation or nitration sites; *d*. The number of predicted sulfation or nitration sites on known phosphorylation residues

	Phosphorylation		Sulfation				Nitration			
Organism	Total*^a^*	Site*^b^*	Total*^c^*	Site*^d^*	E-ratio	*p*-value	Total*^c^*	Site*^d^*	E-ratio	*p*-value
***HS***	126,147	13,730	8,468	1,604	1.74	8.05E-115	13,033	2,051	1.45	1.07E-71
***MM***	93,152	9,378	6,528	1,167	1.78	3.79E-89	9,864	1,463	1.47	2.68E-56
***DM***	17,611	904	1,469	114	1.51	4.57E-06	1,863	140	1.46	1.90E-06
***CE***	4,870	230	550	28	1.08	0.364	546	35	1.36	3.49E-02
**Total**	241,780	24,242	17,015	2,913	1.71	3.04E-192	25,306	3,689	1.45	1.05E-129

**Table 4:** The statistical analysis of human variations that change singly or multiply modified tyrosines. The experimentally identified or predicted tyrosine modification sites were mapped to the HGMD^46^, CanProVar^47^, and SNPs of 1000 Genomes project^54^.*a.* Num., the number of modified tyrosines which can be mapped to public databases; *b.* All, the number of all modified tyrosines

	Phosphorylation		Sulf.-Phos.				Nit.-Phos.			
Resource	Num.*^a^*	All*^b^*	Num.	All	E-ratio	*p*-value	Num.	All	E-ratio	*p*-value
HGMD	31	13730	2	1604	0.55	0.28	6	2051	1.30	0.31
CanProVar	40	13730	3	1604	0.64	0.30	4	2051	0.68	0.27
1000 Genome	64	13730	8	1604	1.07	0.48	12	2051	1.26	0.24
