# Supplementary material for: Systematic analysis of the in situ crosstalk of tyrosine modifications reveals no additional natural selection on multiply modified residues
Source: Sci Rep. 2014 Dec 5;4:7331. doi: 10.1038/srep07331 (PMC4256647; doi:10.1038/srep07331)
Supplement: Supplementary Information — Supplemental experimental procedures and supplemental tables [file srep07331-s1.doc]

# Supplemental materials

# Systematic analysis of the *in situ* crosstalk of tyrosine modifications reveals no additional natural selection on multiply modified residues

Zhicheng Pan^1,†^, Zexian Liu^1,†^, Han Cheng^1^, Tianshun Gao^1^, Shahid Ullah^1^, Jian Ren^2^, and Yu Xue^1^,*

^1^Department of Biomedical Engineering, College of Life Science and Technology, Huazhong University of Science and Technology, Wuhan, Hubei 430074, China

^3^State Key Laboratory of Biocontrol, School of Life Sciences, Sun Yat-sen University, Guangzhou, Guangdong 510275, China

*To whom correspondence may be addressed. Yu Xue, Tel: +86-27-87793903, Fax: +86-27-87793172, E-mail: xueyu@hust.edu.cn.

**Supplemental experimental procedures**

## *The algorithm of GPS-TSP*

For the prediction of sulfation sites, we used the self-developed GPS 2.2 algorithm , which contains two computational parts including a scoring strategy and performance improvement. The basic hypothesis of the scoring strategy is that similarly short peptides would exhibit similar 3D structures and biochemical properties . First, we defined *sulfation site peptide* SSP(*m, n*) as a tyrosine amino acid flanked by *m* residues upstream and *n* residues downstream. Then we used the amino acid substitution matrix BLOSUM62 to estimate the similarity between two SSP(*m*, *n*) peptides *A* and *B* as:

*Score*(*A*[*i*], *B*[*i*]) denotes the substitution score of the two amino acids of *A*[*i*] and *B*[*i*] in the BLOSUM62 at the position *i*. If *S*(*A*, *B*) < 0, we redefined it as *S*(*A*, *B*) = 0. A given SSP(*m, n*) is then compared with each of the experimentally verified sulfated peptides in a pairwise manner to calculate the similarity scores. The average value of the similarity scores is regarded as the final score.

The performance improvement procedure comprises three steps, including motif length selection, weight training and matrix mutation.

1) **Motif length selection**. To optimize the length of the sequence window for prediction, the combinations of SSP(*m*, *n*) (*m* = 1, …, 30; *n* = 1, …, 30) were exhaustively tested, the average *Sn* values were calculated under the *Sp* of 85, 90 and 95% in each time. In this step, the optimal combination of SSP(8, 15) was determined based on the highest average *Sn* value.

2) **Weight training**. With the hypothesis that different positions around the modified tyrosine residues might have different contributions to sulfation, the weights of the every positions in the sequence window were optimized. The substitution score between two SSP(*m*, *n*) peptides *A* and *B* was refined as:

Initially, the weight of each position in SSP(*m*, *n*) was defined as 1. The *w_i_* value is the weight of position *i*. Again, if *S′*(*A*, *B*) < 0, we redefined it as *S′*(*A*, *B*) = 0. Then we randomly picked out the weight of any position for +1 or -1 and re-computed the LOO result. The average *Sn* values were calculated under the *Sp* of 85, 90 and 95%. The manipulation was continued until the *Sn* value was not increased any further.

3) **Matrix mutation**. The aim of this step is to generate an optimal or near-optimal scoring matrix. BLOSUM62 was chosen as the initial matrix, and the LOO performance was calculated. Again, we calculated the average *Sn* with the Sp of 85, 90 and 95%. To improve the average *Sn* value, one element was randomly picked out for +1 or -1. This process was repeated until convergence was reached. Choosing a different initial matrix, e.g., BLOSUM45, will generate a convergent result if the training time is sufficient (Data not shown).

To promise a satisfying result, the training order of three steps was shuffled and repeated several times until the convergence reached. Finally, the SSP(8, 15) was chosen for its best performance of prediction. The GPS-TSP 1.0 was implemented in JAVA, and the SSP(7, 7) is selected to present prediction results in the software for convenience.

**References**

1. Liu, Z., Ma, Q., Cao, J., Gao, X., Ren, J., and Xue, Y. (2011) GPS-PUP: computational prediction of pupylation sites in prokaryotic proteins. *Mol Biosyst* 7, 2737-2740.

2. Liu, Z., Cao, J., Ma, Q., Gao, X., Ren, J., and Xue, Y. (2011) GPS-YNO2: computational prediction of tyrosine nitration sites in proteins. *Molecular bioSystems* 7, 1197-1204.

**Supplemental tables**

**Supplemental Table 1** - Potential *in situ* crosstalks among sulfation, nitration and phosphorylation. From experimentally identified PTM sites, we pairwisely detected the modified sites that can be regulated by two PTMs. *a*. Position, the position of modified tyrosine; *b*. PMID, the primary references for modification.

| **Gene name** | **UniProt** | **Position***^a^* | **Organism** | **PMID***^b^* |
| --- | --- | --- | --- | --- |
| ***The crosstalks between sulfation and nitration*** | | | |  |
| S1pr1 | O08530 | 19 | *M. musculus* | 16148028; 17615369 |
| Hirudin-1 | P01050 | 63 | *H. medicinalis* | 8251938; 1991108 |
| ***The crosstalks between phosphorylation and sulfation*** | | | | |
| GAST | P01350 | 87 | *H. sapiens* | 15212693; 17962309; 19638616; 7530658 |
| C5AR1 | P21730 | 11 | *H. sapiens* | 15174125; 11342590 |
| C5AR1 | P21730 | 14 | *H. sapiens* | 15174125; 11342590 |
| ***The crosstalks between phosphorylation and nitration*** | | | | |
| PDLIM1 | O00151 | 321 | *H. sapiens* | 15212693; 17962309; 19638616; 15174125; 19366988; 18988627; 19741252 |
| Dpysl2 | O08553 | 431 | *M. musculus* | 15174125; 19366988; 17497906 |
| BUB3 | O43684 | 207 | *H. sapiens* | 19638616; 19741252 |
| PRPSAP2 | O60256 | 52 | *H. sapiens* | 19638616; 15174125; 19741252 |
| SYNCRIP | O60506 | 373 | *H. sapiens* | 15212693; 17962309; 19638616; 15174125; 19741252 |
| TBCA | O75347 | 94 | *H. sapiens* | 19638616; 19741252 |
| SEC22B | O75396 | 33 | *H. sapiens* | 15174125; 19741252 |
| RNASEH2A | O75792 | 172 | *H. sapiens* | 19638616; 15174125; 19741252 |
| PSMD10 | O75832 | 138 | *H. sapiens* | 19638616; 15174125; 19741252 |
| PROSC | O94903 | 69 | *H. sapiens* | 19638616; 19060867; 15174125; 19741252 |
| LDHA | P00338 | 10 | *H. sapiens* | 19638616; 15174125; 19741252 |
| LDHA | P00338 | 239 | *H. sapiens* | 15212693; 17962309; 19638616; 19060867; 15174125; 18988627; 19741252 |
| PGK1 | P00558 | 76 | *H. sapiens* | 19638616; 15174125; 19741252; 17497906 |
| PGK1 | P00558 | 196 | *H. sapiens* | 15212693; 17962309; 19638616; 19060867; 15174125; 18988627; 19741252 |
| C5 | P01031 | 700 | *H. sapiens* | 19060867; 2410424 |
| COL4A1 | P02462 | 348 | *H. sapiens* | 19638616; 15174125; 17334231 |
| SOD2 | P04179 | 58 | *H. sapiens* | 19638616; 9484232; 9603906; 10334867; 16443160 |
| Mbp | P04370 | 199 | *M. musculus* | 15174125; 19366988; 17497906 |
| Aldoa | P05064 | 5 | *M. musculus* | 15174125; 17497906 |
| SLC25A5 | P05141 | 195 | *H. sapiens* | 19638616; 15174125; 19741252 |
| Got1 | P05201 | 71 | *M. musculus* | 15174125; 19366988; 17497906 |
| Got2 | P05202 | 96 | *M. musculus* | 15174125; 19366988; 17497906 |
| NPM1 | P06748 | 67 | *H. sapiens* | 15212693; 17962309; 15174125; 19366988; 18988627; 19741252 |
| ANXA2 | P07355 | 30 | *H. sapiens* | 15212693; 17962309; 19638616; 19060867; 15174125; 19366988; 18988627; 19741252 |
| Alb | P07724 | 365 | *M. musculus* | 15174125; 17615369 |
| PFN1 | P07737 | 60 | *H. sapiens* | 19638616; 15174125; 19741252 |
| PFN1 | P07737 | 129 | *H. sapiens* | 15212693; 17962309; 19638616; 19060867; 15174125; 18988627; 19741252 |
| HSP90AA1 | P07900 | 197 | *H. sapiens* | 19638616; 15174125; 17497906 |
| HNRNPC | P07910 | 139 | *H. sapiens* | 19638616; 15174125; 19741252 |
| VIM | P08670 | 30 | *H. sapiens* | 19638616; 15174125; 19366988; 18988627; 19741252 |
| VIM | P08670 | 276 | *H. sapiens* | 19638616; 15174125; 19741252 |
| HMGB1 | P09429 | 78 | *H. sapiens* | 19638616; 15174125; 19741252 |
| Gstm1 | P10649 | 23 | *M. musculus* | 15174125; 19366988; 11594736 |
| Gstm1 | P10649 | 28 | *M. musculus* | 15174125; 11594736 |
| Gstm1 | P10649 | 33 | *M. musculus* | 15212693; 17962309; 11594736 |
| HSPD1 | P10809 | 243 | *H. sapiens* | 19638616; 15174125; 19741252 |
| Hist1h2bf | P10853 | 38 | *M. musculus* | 15174125; 11723112 |
| Hist1h2bf | P10853 | 41 | *M. musculus* | 15174125; 11723112 |
| Hist1h2bf | P10853 | 43 | *M. musculus* | 15174125; 19366988; 11723112; 19741252 |
| HSPA8 | P11142 | 134 | *H. sapiens* | 19638616; 15174125; 19741252; 16800626 |
| G6PD | P11413 | 401 | *H. sapiens* | 15212693; 17962309; 19638616; 19060867; 15174125; 18988627; 19741252 |
| EEF2 | P13639 | 443 | *H. sapiens* | 19638616; 19741252 |
| LCP1 | P13796 | 598 | *H. sapiens* | 19638616; 15174125; 19741252 |
| Rpsa | P14206 | 139 | *M. musculus* | 15212693; 17962309; 15174125; 19366988; 16800626 |
| PKM2 | P14618 | 105 | *H. sapiens* | 15212693; 17962309; 19638616; 15174125; 17497906 |
| PKM2 | P14618 | 148 | *H. sapiens* | 15174125; 19741252; 15851474 |
| HSP90B1 | P14625 | 677 | *H. sapiens* | 19638616; 19060867; 15174125; 18988627; 19741252 |
| MYL6B | P14649 | 86 | *H. sapiens* | 19060867; 19741252 |
| EZR | P15311 | 424 | *H. sapiens* | 19638616; 15174125; 19741252 |
| RPS2 | P15880 | 266 | *H. sapiens* | 19638616; 15174125; 19366988; 19741252 |
| PECAM1 | P16284 | 713 | *H. sapiens* | 15212693; 17962309; 19060867; 15174125; 19366988; 18988627; 12207897 |
| HIST1H1B | P16401 | 74 | *H. sapiens* | 19060867; 15174125; 17497906; 19741252 |
| Gapdh | P16858 | 316 | *M. musculus* | 15212693; 17962309; 15174125; 19366988; 17497906 |
| Tpi1 | P17751 | 68 | *M. musculus* | 15174125; 16800626 |
| Tpi1 | P17751 | 165 | *M. musculus* | 15174125; 17497906 |
| Tpi1 | P17751 | 209 | *M. musculus* | 15174125; 16800626 |
| PGAM1 | P18669 | 92 | *H. sapiens* | 19638616; 19060867; 15174125; 18988627; 17497906 |
| NCL | P19338 | 351 | *H. sapiens* | 19638616; 19741252 |
| NCL | P19338 | 525 | *H. sapiens* | 19638616; 19741252 |
| CSNK2A2 | P19784 | 13 | *H. sapiens* | 19638616; 15174125; 19741252 |
| LMNB1 | P20700 | 482 | *H. sapiens* | 19638616; 19741252 |
| FLNA | P21333 | 373 | *H. sapiens* | 19060867; 15174125; 18988627; 19741252 |
| FLNA | P21333 | 1308 | *H. sapiens* | 19638616; 19741252 |
| Fgfr2 | P21803 | 656 | *M. musculus* | 15174125; 17898259; |
| PAICS | P22234 | 22 | *H. sapiens* | 19638616; 19060867; 15174125; 19366988; 18988627; 19741252 |
| TCEA1 | P23193 | 126 | *H. sapiens* | 19638616; 15174125; 19366988; 19741252 |
| SFPQ | P23246 | 691 | *H. sapiens* | 19638616; 15174125; 19741252 |
| CFL1 | P23528 | 68 | *H. sapiens* | 15212693; 17962309; 19638616; 19060867; 15174125; 18988627; 19741252; 17497906 |
| CFL1 | P23528 | 140 | *H. sapiens* | 15212693; 17962309; 19638616; 19060867; 15174125; 19366988; 18988627; 19741252 |
| ACP1 | P24666 | 88 | *H. sapiens* | 19638616; 19741252 |
| PSMA3 | P25788 | 8 | *H. sapiens* | 15174125; 19366988; 19741252 |
| NFKBIA | P25963 | 305 | *H. sapiens* | 15212693; 17962309; 19638616; 15174125; 17910475 |
| PTBP1 | P26599 | 430 | *H. sapiens* | 19638616; 19741252 |
| TARS | P26639 | 298 | *H. sapiens* | 19638616; 19060867; 15174125; 19741252 |
| MAP4 | P27816 | 1001 | *H. sapiens* | 19638616; 15174125; 19366988; 19741252 |
| PSMB6 | P28072 | 59 | *H. sapiens* | 19638616; 15174125; 19741252 |
| NOS3 | P29474 | 81 | *H. sapiens* | 19060867; 15174125; 18988627; 18756022 |
| NOS3 | P29474 | 657 | *H. sapiens* | 15174125; 18756022 |
| EEF1D | P29692 | 26 | *H. sapiens* | 19638616; 15174125; 19741252 |
| PEBP1 | P30086 | 64 | *H. sapiens* | 19638616; 19741252 |
| STIP1 | P31948 | 354 | *H. sapiens* | 15212693; 17962309; 19638616; 15174125; 18988627; 19741252 |
| PFN2 | P35080 | 99 | *H. sapiens* | 19638616; 19060867; 15174125; 18988627; 19741252 |
| FUS | P35637 | 468 | *H. sapiens* | 19638616; 15174125; 19741252 |
| GMPR | P36959 | 318 | *H. sapiens* | 19638616; 19741252 |
| TAGLN2 | P37802 | 192 | *H. sapiens* | 19638616; 19060867; 15174125; 19741252 |
| SNCA | P37840 | 39 | *H. sapiens* | 15174125; 11062131; 12031537; 19697948; 15699043; 17497906 |
| SNCA | P37840 | 125 | *H. sapiens* | 15212693; 17962309; 19638616; 15174125; 18988627; 11062131; 12031537; 19697948 |
| SNCA | P37840 | 133 | *H. sapiens* | 15212693; 17962309; 19638616; 15174125; 18988627; 11062131; 12031537; 19697948 |
| SNCA | P37840 | 136 | *H. sapiens* | 15212693; 17962309; 19638616; 15174125; 18988627; 11062131; 12031537; 19697948 |
| Dnm1 | P39053 | 125 | *M. musculus* | 15174125; 19366988; 16800626 |
| Dnm1 | P39053 | 354 | *M. musculus* | 15212693; 17962309; 15174125; 19366988; 17497906 |
| Tkt | P40142 | 275 | *M. musculus* | 15212693; 17962309; 15174125; 19366988; 17497906 |
| MDH2 | P40926 | 56 | *H. sapiens* | 15212693; 17962309; 19638616; 15174125; 18988627; 17497906; 18708664 |
| MDH2 | P40926 | 80 | *H. sapiens* | 19638616; 18708664 |
| MDH2 | P40926 | 161 | *H. sapiens* | 19638616; 19741252 |
| MATR3 | P43243 | 202 | *H. sapiens* | 19638616; 15174125; 18988627; 19741252 |
| RPL34 | P49207 | 13 | *H. sapiens* | 19638616; 15174125; 19366988; 19741252 |
| CCT4 | P50991 | 269 | *H. sapiens* | 19638616; 19741252 |
| HNRNPM | P52272 | 64 | *H. sapiens* | 19638616; 15174125; 19741252 |
| ARHGDIB | P52566 | 24 | *H. sapiens* | 15212693; 17962309; 19638616; 19060867; 15174125; 18988627; 19741252 |
| ACLY | P53396 | 384 | *H. sapiens* | 19638616; 19741252 |
| VCP | P55072 | 644 | *H. sapiens* | 19638616; 15174125; 19741252 |
| EIF3B | P55884 | 449 | *H. sapiens* | 19638616; 15174125; 19741252 |
| Plp1 | P60202 | 207 | *M. musculus* | 15174125; 18708664 |
| Actb | P60710 | 91 | *M. musculus* | 15174125; 19366988; 12401783; 17497906 |
| Actb | P60710 | 169 | *M. musculus* | 15174125; 19366988; 17497906 |
| Actb | P60710 | 198 | *M. musculus* | 15174125; 19366988; 12401783; 19741252 |
| Actb | P60710 | 362 | *M. musculus* | 15174125; 12401783; 19741252 |
| EIF4A1 | P60842 | 70 | *H. sapiens* | 19638616; 19741252 |
| EIF4A1 | P60842 | 197 | *H. sapiens* | 19638616; 15174125; 19741252 |
| ACTR3 | P61158 | 16 | *H. sapiens* | 19638616; 19060867; 15174125; 18988627; 19741252 |
| ABCE1 | P61221 | 594 | *H. sapiens* | 19638616; 19741252 |
| HSPE1 | P61604 | 88 | *H. sapiens* | 19638616; 19060867; 15174125; 18988627; 19741252 |
| HNRNPK | P61978 | 72 | *H. sapiens* | 19638616; 15174125; 18988627; 19741252 |
| HNRNPK | P61978 | 323 | *H. sapiens* | 19638616; 15174125; 19741252 |
| CALM1 | P62158 | 100 | *H. sapiens* | 15212693; 17962309; 19638616; 19060867; 15174125; 19366988; 18988627; 12693036; 204335 |
| CALM1 | P62158 | 139 | *H. sapiens* | 15174125; 18988627; 12693036; 8011644; 204335; 212099 |
| RPS8 | P62241 | 83 | *H. sapiens* | 19638616; 15174125; 19741252 |
| RPS8 | P62241 | 188 | *H. sapiens* | 15174125; 19741252 |
| Ywhae | P62259 | 214 | *M. musculus* | 15174125; 17497906; 19741252 |
| RPS11 | P62280 | 55 | *H. sapiens* | 15174125; 19741252 |
| HIST1H4A | P62805 | 52 | *H. sapiens* | 15212693; 17962309; 19638616; 19060867; 15174125; 19741252; 11723112; 17497906 |
| HIST1H4A | P62805 | 73 | *H. sapiens* | 19638616; 19060867; 15174125; 19741252; 11723112; 17497906 |
| HIST1H4A | P62805 | 89 | *H. sapiens* | 19638616; 19060867; 15174125; 19741252; 11723112; 17497906 |
| RAN | P62826 | 147 | *H. sapiens* | 15212693; 17962309; 19638616; 15174125; 18988627; 19741252 |
| RPL8 | P62917 | 133 | *H. sapiens* | 15212693; 17962309; 19638616; 15174125; 18988627; 19741252 |
| GRB2 | P62993 | 209 | *H. sapiens* | 15212693; 17962309; 19638616; 15174125; 19366988; 18988627; 19741252 |
| GNB2L1 | P63244 | 52 | *H. sapiens* | 19638616; 19741252 |
| Phb | P67778 | 249 | *M. musculus* | 15174125; 17497906 |
| EEF1A1 | P68104 | 29 | *H. sapiens* | 15212693; 17962309; 19638616; 19060867; 15174125; 18988627; 19741252 |
| EEF1A1 | P68104 | 254 | *H. sapiens* | 19638616; 15174125; 19366988; 19741252 |
| HBB | P68871 | 131 | *H. sapiens* | 19638616; 19060867; 15174125; 18988627; 10828987; 11735412; 18161731 |
| HBB | P68871 | 146 | *H. sapiens* | 19638616; 19060867; 15174125; 17497906 |
| HBA1 | P69905 | 25 | *H. sapiens* | 19638616; 19060867; 15174125; 18988627; 10828987; 11735412; 18161731; 17497906 |
| HBA1 | P69905 | 43 | *H. sapiens* | 19638616; 19060867; 15174125; 18988627; 10828987; 11735412; 18161731; 17497906; 18708664 |
| SRSF3 | P84103 | 32 | *H. sapiens* | 19638616; 15174125; 19741252 |
| SLC25A3 | Q00325 | 196 | *H. sapiens* | 15174125; 19741252 |
| CLTC | Q00610 | 883 | *H. sapiens* | 19638616; 15174125; 19366988; 19741252 |
| CAP1 | Q01518 | 419 | *H. sapiens* | 19638616; 19741252 |
| Ckb | Q04447 | 39 | *M. musculus* | 15174125; 19366988; 15699043; 17497906 |
| SSBP1 | Q04837 | 73 | *H. sapiens* | 15212693; 17962309; 19638616; 19060867; 15174125; 19741252 |
| PRDX1 | Q06830 | 194 | *H. sapiens* | 15212693; 17962309; 19638616; 15174125; 18988627; 19741252 |
| SRSF1 | Q07955 | 37 | *H. sapiens* | 19638616; 15174125; 19741252 |
| SRSF1 | Q07955 | 170 | *H. sapiens* | 19638616; 15174125; 19741252 |
| SRSF1 | Q07955 | 189 | *H. sapiens* | 19638616; 19060867; 15174125; 18988627; 19741252 |
| NSUN2 | Q08J23 | 646 | *H. sapiens* | 15174125; 19741252 |
| ILF3 | Q12906 | 579 | *H. sapiens* | 15174125; 19366988; 19741252 |
| MAD2L1 | Q13257 | 199 | *H. sapiens* | 19638616; 19741252 |
| TRIM28 | Q13263 | 242 | *H. sapiens* | 19638616; 15174125; 19741252 |
| TRIM28 | Q13263 | 517 | *H. sapiens* | 19638616; 19741252 |
| EIF3I | Q13347 | 308 | *H. sapiens* | 19638616; 19060867; 15174125; 18988627; 19741252 |
| HDAC1 | Q13547 | 87 | *H. sapiens* | 19638616; 19741252 |
| NACA | Q13765 | 120 | *H. sapiens* | 19638616; 15174125; 19741252 |
| SPTAN1 | Q13813 | 942 | *H. sapiens* | 19638616; 17497906 |
| SPTAN1 | Q13813 | 976 | *H. sapiens* | 19638616; 19741252 |
| SPTAN1 | Q13813 | 2423 | *H. sapiens* | 19638616; 19060867; 15174125; 18988627; 18708664 |
| EIF4H | Q15056 | 12 | *H. sapiens* | 15212693; 17962309; 19638616; 15174125; 19366988; 18988627; 19741252 |
| EIF4H | Q15056 | 86 | *H. sapiens* | 15174125; 19741252 |
| EIF4H | Q15056 | 101 | *H. sapiens* | 15212693; 17962309; 19638616; 15174125; 18988627; 19741252 |
| PCBP1 | Q15365 | 183 | *H. sapiens* | 19638616; 19366988; 18988627; 19741252 |
| SF3B4 | Q15427 | 16 | *H. sapiens* | 19638616; 19060867; 15174125; 18988627; 19741252 |
| SF3A1 | Q15459 | 456 | *H. sapiens* | 15212693; 17962309; 19638616; 15174125; 18988627; 19741252 |
| HSP90AB2P | Q58FF8 | 260 | *H. sapiens* | 15174125; 19741252 |
| SND1 | Q7KZF4 | 329 | *H. sapiens* | 15174125; 19741252 |
| Tubb2a | Q7TMM9 | 36 | *M. musculus* | 15174125; 19366988; 17497906 |
| Actr1b | Q8R5C5 | 4 | *M. musculus* | 15174125; 16800626 |
| UPF1 | Q92900 | 946 | *H. sapiens* | 19638616; 15174125; 19741252 |
| RBM14 | Q96PK6 | 273 | *H. sapiens* | 19638616; 15174125; 19741252 |
| Atp5o | Q9DB20 | 35 | *M. musculus* | 15174125; 19366988; 17497906 |
| NANS | Q9NR45 | 71 | *H. sapiens* | 19638616; 19741252 |
| ACTR10 | Q9NZ32 | 4 | *H. sapiens* | 19638616; 19741252 |
| IL36A | Q9UHA7 | 96 | *H. sapiens* | 19638616; 15174125; 16777052 |
| CHORDC1 | Q9UHD1 | 292 | *H. sapiens* | 19638616; 15174125; 19741252 |
| CDV3 | Q9UKY7 | 95 | *H. sapiens* | 15212693; 17962309; 19638616; 15174125; 19366988; 18988627; 19741252 |
| NSFL1C | Q9UNZ2 | 167 | *H. sapiens* | 19638616; 15174125; 19366988; 19741252 |
| RUVBL2 | Q9Y230 | 430 | *H. sapiens* | 19638616; 19741252 |
| STRAP | Q9Y3F4 | 114 | *H. sapiens* | 19638616; 15174125; 19741252 |
| LDHB | P07195 | 84 | *H. sapiens* | 19638616; 19741252 |
| LDHB | P07195 | 240 | *H. sapiens* | 15212693; 17962309; 19638616; 19060867; 15174125; 18988627; 19741252; 7092806 |
| HRAS | P01112 | 157 | *H. sapiens* | 19638616; 16841939 |
| PPP2CA | P67775 | 284 | *H. sapiens* | 19638616; 15174125; 20100830 |
| TP53 | P04637 | 327 | *H. sapiens* | 19638616; 20499882 |
| MYL3 | P08590 | 73 | *H. sapiens* | 19638616; 20518849 |

**Supplemental Table 2** - The summarization of predicted sulfation and nitration sites on known phosphorylated substrates collected in this study. a. The number of known phosphorylated substrates; b. The number of the tyrosines on the substrates. c. The phosphorylation sites might also be modified by either sulfation or nitration.

| **Organism** | **Phosphorylation** | | | **Sulfation** | | | | **Nitration** | | | | **Either***^c^* | | | |
| --- | --- | --- | --- | --- | --- | --- | --- | --- | --- | --- | --- | --- | --- | --- | --- |
|  | **Sub.***^a^* | **Total***^b^* | **Site** | **Total** | **Site** | **E-ratio** | ***p*-value** | **Total** | **Site** | **E-ratio** | ***p*-value** | **Total** | **Site** | **E-ratio** | ***p*-value** |
| ***H. sapiens*** | 5,876 | 126,147 | 13,730 | 8,468 | 1,604 | 1.74 | 8.05E-115 | 13,033 | 2,051 | 1.45 | 1.07E-71 | 20,033 | 3,299 | 1.51 | 1.12E-152 |
| ***M. musculus*** | 4,302 | 93,152 | 9,378 | 6,528 | 1,167 | 1.78 | 3.72E-115 | 9,864 | 1,463 | 1.47 | 2.68E-56 | 15,268 | 2,355 | 1.53 | 1.37E-115 |
| ***D. melanogaster*** | 657 | 17,611 | 904 | 1,469 | 114 | 1.51 | 4.57E-06 | 1,863 | 140 | 1.46 | 1.90E-06 | 3,106 | 228 | 1.43 | 2.67E-09 |
| ***C. elegans*** | 199 | 4,870 | 230 | 550 | 28 | 1.08 | 0.364 | 546 | 35 | 1.36 | 0.035 | 1,013 | 57 | 1.19 | 0.077 |
| **Total** | 11,034 | 241,780 | 24,242 | 17,015 | 2,913 | 1.71 | 1.55E-215 | 25,306 | 3,689 | 1.45 | 1.05E-129 | 39,420 | 5,939 | 1.50 | 4.10E-263 |

**Supplemental Table 3** - The summarization of predicted sulfation and nitration sites on known phosphorylated substrates from Phospho.ELM. a. The number of the tyrosines on the substrates. b. The phosphorylation sites might also be modified by either sulfation or nitration.

| **Organism** | **Phosphorylation** | | **Sulfation** | | | | **Nitration** | | | | **Eitherb** | | | |
| --- | --- | --- | --- | --- | --- | --- | --- | --- | --- | --- | --- | --- | --- | --- |
|  | **Totala** | **p-site** | **Total** | **p-site** | **E-ratio** | ***p-value*** | **Total** | **p-site** | **E-ratio** | ***p-value*** | **Total** | **p-site** | **E-ratio** | ***p-value*** |
| ***H. sapiens*** | 28,304 | 2,394 | 2,317 | 407 | 2.08 | 2.43E-49 | 3,272 | 456 | 1.65 | 3.48E-29 | 5,193 | 758 | 1.73 | 3.97E-61 |
| ***M. musculus*** | 8,528 | 665 | 759 | 137 | 2.31 | 1.77E-22 | 1,011 | 134 | 1.70 | 1.02E-10 | 1,639 | 233 | 1.82 | 7.55E-24 |
| ***D. melanogaster*** | 1,533 | 84 | 150 | 17 | 2.07 | 2.17E-03 | 191 | 22 | 2.10 | 3.25E-04 | 312 | 32 | 1.87 | 8.73E-05 |
| ***C. elegans*** | 2,236 | 111 | 260 | 12 | 0.93 | 4.65E-01 | 287 | 15 | 1.05 | 4.58E-01 | 486 | 24 | 0.99 | 5.43E-01 |
| **Total** | 40,601 | 3,254 | 3,486 | 573 | 2.05 | 1.60E-66 | 4,761 | 627 | 1.64 | 6.57E-39 | 7,630 | 1,047 | 1.71 | 1.63E-81 |

**Supplemental Table 4** - The in situ crosstalk sites which can be mapped to HGMD. a. The in situ crosstalk sites of sulfation and phosphorylation. b. The in situ crosstalk sites of nitration and phosphorylation.

| **Gene name** | **UniProt** | **Mutation** | **SNP** | **HGMD** | **Disease/phenotype** | **PMID** |
| --- | --- | --- | --- | --- | --- | --- |
| ***Sulfation-phosphorylation^a^*** | | | | | | |
| PTPN11 | Q06124 | Y63C | rs121918459 | CM013416 | Noonan syndrome | 11704759 |
| PTPN11 | Q06124 | Y62D | rs121918460 | CM021128 | Noonan syndrome | 11992261 |
| PTPN11 | Q06124 | Y62N | rs121918460 | CM060446 | Noonan syndrome | 16358218 |
| ***Nitration-phosphorylation^b^*** | | | | | | |
| CYP17A1 | P05093 | Y329D | rs104894144 | CM033601 | Steroid-17 alpha-hydroxylase deficiency | 14671162 |
| FAS | P25445 | Y232C | rs121913079 | CM971494 | Autoimmune lymphoproliferative syndrome | 9028321 |
| CSF1R | P07333 | Y969C | rs1801271 | CM900079 | Myleoid malignancy, predisposition | 2406720 |
| HBA1 | P69905 | Y25C | rs28928880 | CM940887 | Haemoglobin variant | 7852094 |
| HBB | P68871 | Y131C | rs33937535 | CM055961 | Erythrocytosis | 15727901 |
| HBB | P68871 | Y131S | rs33937535 | CM900314 | Haemoglobin variant | 2384309 |
| GFAP | P14136 | Y242D | rs60551555 | CM023073 | Alexander disease | 12034785 |

**Supplemental Table 5** - The in situ crosstalk sites which can be mapped to CanProVar. a. The in situ crosstalk sites of sulfation and phosphorylation. b. The in situ crosstalk sites of nitration and phosphorylation.

| **Gene name** | **UniProt** | **Variations** | **CanProVar** | **Cancer sample** | **PMID** |
| --- | --- | --- | --- | --- | --- |
| ***Sulfation-phosphorylation^a^*** | | | | | |
| KIT | P10721 | Y568C | cs2449 | soft_tissue cancer | 10665649; 18428421 |
| KIT | P10721 | Y568D | cs2432 | soft_tissue cancer | 10485475; 14694524; 18428421 |
| KIT | P10721 | Y568S | cs9585 | soft_tissue cancer | 18428421; 19411681 |
| PTPN11 | Q06124 | Y63C | cs3721 | haematopoietic_and_lymphoid_tissue cancer | 14644997; 18428421 |
| IMP4 | Q96G21 | Y226H | cs10967 | entral_nervous_system cancer | 18428421; 18772396 |
| ***Nitration-phosphorylation^b^*** | | | | | |
| PRKRA | O75569 | Y270* | cs10106 | skin cancer | 18428421; 20016485 |
| TP53 | P04637 | Y327S | cs5268 | sporadic cancer | 1905840; 8829653 |
| TP53 | P04637 | Y327H | cs6383 | sporadic cancer | 1905840; 8829653 |
| RB1 | P06400 | Y325* | cs8541 | eye cancer, urinary_tract cancer | 12955724; 18428421 |
| CSF1R | P07333 | Y969H | cs479 | haematopoietic_and_lymphoid_tissue cancer | 18428421; 2406720; 7885045 |
| CSF1R | P07333 | Y969N | cs480 | haematopoietic_and_lymphoid_tissue cancer | 18428421; 2406720 |
| CSF1R | P07333 | Y969F | cs477 | haematopoietic_and_lymphoid_tissue cancer | 18428421; 2142747; 2406720 |
| CSF1R | P07333 | Y969C | cs484 | haematopoietic_and_lymphoid_tissue cancer | 18428421; 2142747; 2406720 |
| CSF1R | P07333 | Y969* | cs486 | haematopoietic_and_lymphoid_tissue cancer | 18428421; 2142747; 2406720 |
| CSF1R | P07333 | Y969D | cs485 | haematopoietic_and_lymphoid_tissue cancer | 18428421; 2406720 |
